# Ethyl pyruvate ameliorates hepatic injury following blunt chest trauma and hemorrhagic shock by reducing local inflammation, NF-kappaB activation and HMGB1 release

**DOI:** 10.1371/journal.pone.0192171

**Published:** 2018-02-08

**Authors:** Nils Wagner, Scott Dieteren, Niklas Franz, Kernt Köhler, Katharina Mörs, Luka Nicin, Julia Schmidt, Mario Perl, Ingo Marzi, Borna Relja

**Affiliations:** 1 Department of Trauma Surgery, University Hospital Frankfurt, Goethe-University, Frankfurt, Germany; 2 Institute of Veterinary Pathology, Justus Liebig University Giessen, Giessen, Germany; 3 BG-Trauma Center Murnau, Murnau, Germany; Medical College of Georgia, Augusta, UNITED STATES

## Abstract

**Background:**

The treatment of patients with multiple trauma including blunt chest/thoracic trauma (TxT) and hemorrhagic shock (H) is still challenging. Numerous studies show detrimental consequences of TxT and HS resulting in strong inflammatory changes, organ injury and mortality. Additionally, the reperfusion (R) phase plays a key role in triggering inflammation and worsening outcome. Ethyl pyruvate (EP), a stable lipophilic ester, has anti-inflammatory properties. Here, the influence of EP on the inflammatory reaction and liver injury in a double hit model of TxT and H/R in rats was explored.

**Methods:**

Female Lewis rats were subjected to TxT followed by hemorrhage/H (60 min, 35±3 mm Hg) and resuscitation/R (TxT+H/R). Reperfusion was performed by either Ringer`s lactated solution (RL) alone or RL supplemented with EP (50 mg/kg). Sham animals underwent all surgical procedures without TxT+H/R. After 2h, blood and liver tissue were collected for analyses, and survival was assessed after 24h.

**Results:**

Resuscitation with EP significantly improved haemoglobin levels and base excess recovery compared with controls after TxT+H/R, respectively (p<0.05). TxT+H/R-induced significant increase in alanine aminotransferase levels and liver injury were attenuated by EP compared with controls (p<0.05). Local inflammation as shown by increased gene expression of IL-6 and ICAM-1, enhanced ICAM-1 and HMGB1 protein expression and infiltration of the liver with neutrophils were also significantly attenuated by EP compared with controls after TxT+H/R (p<0.05). EP significantly reduced TxT+H/R-induced p65 activation in liver tissue. Survival rates improved by EP from 50% to 70% after TxT+H/R.

**Conclusions:**

These data support the concept that the pronounced local pro-inflammatory response in the liver after blunt chest trauma and hemorrhagic shock is associated with NF-κB. In particular, the beneficial anti-inflammatory effects of ethyl pyruvate seem to be regulated by the HMGB1/NF-κB axis in the liver, thereby, restraining inflammatory responses and liver injury after double hit trauma in the rat.

## Introduction

Trauma is still the most common cause of death in children and young adults, as well as one of the global leading causes of worldwide mortality [1,2]. The treatment of patients with severe and multiple traumatic injuries including blunt chest trauma and hemorrhagic shock is still challenging [3,4]. Due to the microcirculatory disturbances and the release of pathogen-associated-molecular-patterns (PAMP) e.g. lipopolysaccharide (LPS) and/or damage-associated-molecular-patterns (DAMP) such as HMGB1 caused by tissue damage, notably these hypoxic conditions result in a massive activation of the immune system, which carries a high risk for multiple organ dysfunction syndrome (MODS) and organ failure (e.g. lung, liver) [5–12].

The local inflammation in the liver during and after hypoxia induces a disturbed glycocalyx function with release of intercellular adhesion molecule (ICAM)-1 and vascular cell adhesion molecule (VCAM)-1, as well as the activation and immigration of monocytes and polymorphonuclear leukocytes (PMNL) into tissue leading to organ damage [13–16]. Subsequent capillary leakage amplifies tissue edema formation and both, tissue hypo-perfusion and hypo-oxygenation [13]. Activation of NF-κB accompanies these pathophysiological processes, and further enhances the production and release of pro-inflammatory mediators including cytokines and HMGB1 [17–19]. The therapeutic strategy with fluid resuscitation is indisputable, however, the required resuscitation after hemorrhagic shock may also trigger further inflammation [13,20] *inter alia* by the release of reactive oxygen and nitrogen species, respectively (ROS and RNS) [21], underlining the importance of an adequate and organ-protective resuscitation strategy [10].

Ethyl pyruvate (EP), the ester formed from ethanol linked to pyruvate, has exerted inflammation-suppressing characteristics in numerous *in vitro* and *in vivo* studies [22]. In our own studies, EP reduced interleukin (IL)-8 release, adhesion of isolated neutrophils and CD54 expression in alveolar epithelial cells in an *in vitro* model of acute inflammation [23]. In an *in vivo* model of ruptured abdominal aortic aneurysma, EP reduced the levels of serum myeloperoxidase, tumor necrosis factor alpha (TNF-α) and the damage of lung tissue compared to control group [24]. Also with regard to brain injuries, EP improved the neurological outcome by reducing inflammation *via* IL-1β and TNF-α, as well as microglia activation [25]. These findings were confirmed in hepatic ischemia-reperfusion injury, where EP reduced alanine aminotransferase (ALT), aspartate aminotransferase (AST), and IL-6 as well as TNF-α levels [26]. Yang *et al*. (2016) provides a nice review about the anti-inflammatory and protective potential of EP in different models, including pancreas, liver, lung, kidney, heart and brain injuries [27].

To the best of our knowledge, no studies have been performed focussing on EP as a therapeutic reperfusion solution after trauma and concomitant massive blood loss. Therefore, based on above shown data, we examined the hypothesis that EP has a beneficial influence on local inflammatory response and liver injury in a clinically relevant double hit trauma model of blunt chest trauma and hemorrhagic shock.

## Material and methods

### Ethics

All experiments and procedures were authorized by the responsible government authority ("Regierungspraesidium Darmstadt, Veterinaerswesen", Hessen, Germany; Nr. of the ethical approval.: FK/1028), and performed in compliance with the federal German law paying attention to the protection of animals, and Institutional Guidelines and the criteria in “Guide for the Care and Use of Laboratory Animals” (Eighth Edition The National Academies Press, 2011) were followed [28]. In our study, we handled the animals permanently in accordance with the ARRIVE guidelines [29]. Animal experiments were performed at the Central Facility of the University Hospital Frankfurt, Goethe-University, Germany.

### Animals and experimental model

LEWIS rats (female, 190–240 g, Janvier Labs, France) were anaesthetised with isoflurane (1.2–3.0%). Buprenorphine (0.05 mg/kg body weight) and a local anaesthesia (0.25% Carbostesin) were applied. The animals were shaved in the area of abdomen, chest, right inguinal and the neck. The right femoral artery was cannulated with polyethylene tubing for blood pressure measurement and blood removal for blood gas analyses (BGA). Subsequently, the bilateral lung contusion was carried out. Here, the animals were placed in supine position and a standardized pressure wave ruptured a Mylar polyester film (0.190 mm DuPont Teijin Films Luxembourg) fixed in a cylinder. The blast wave was directed to the thorax of the animals [30,31]. After a short stabilization phase, cannulation of the left jugular vein and the right carotid artery was performed. Then, hemorrhagic shock was initiated by taking stepwise blood *via* the carotid artery until a mean arterial blood pressure (MABP) of 35 ± 3 mm Hg was reached. This blood pressure was kept constant for 60 min using a blood pressure analyzer (Siemens AG), if necessary by further withdrawal or recirculation of withdrawn blood [16,32]. At the end of the hemorrhagic shock period reperfusion was carried out *via* the jugular vein. Depending on the group assignment, the reperfusion was performed by blood and Ringer’s lactated solution (RL), or blood and RL supplemented with EP. 60% of the withdrawn blood volume plus 50% RL or 50% RL plus EP (Sigma Aldrich, 50 mg/kg body weight) of the maximum removed volume were reperfused. After completion of a 30 minutes reperfusion period the catheters were removed, the wounds were closed and anesthesia was terminated. During the experimental period a continuous temperature monitoring was performed.

In one experimental arm, two hours after end of the experiment, sacrifice was performed. Under isoflurane anesthesia the abdominal aorta was punctured by a laparotomy and blood was withdrawn. Flushing was carried out *via* the *V*. *cava* using 20 ml RL. The left liver flap was removed and snap-frozen in liquid nitrogen. Finally, rinsing by 20 ml of 10% buffered formalin solution and the removal of the right liver lobe followed. The right liver lobe was enclosed in paraffin and during the further procedure sectioned and stained with hematoxylin-eosin.

In the other experimental arm, survival was assessed after 24 hours.

### Animal welfare/ monitoring

Acclimation of the animals was ensured for at least 7 days. Experimentation was performed in the same building to keep the stress level of the animals as low as possible.

Anaesthesia was performed as above described. Buprenorphine (0.05 mg/kg body weight) and a local anaesthesia (0.25% Carbostesin) were applied. The first application of Buprenorphine was conducted 30 min prior experimentation. Postoperative Buprenorphine was given twice daily until the end of experimentation. During the complete experimentation period a continuous monitoring of blood pressure and temperature was performed. The animals received free access to water and food postoperative. After the experimentation, their condition has been controlled, and euthanasia (of all animals, which were grouped to “died”) was performed in case they met the requirements in accordance with the guidelines of the Ethics Committee of the RP Darmstadt as described above. All involved group members hold the certificate of the Federation of European Laboratory Animal Science Associations (FELASA).

### Group allocation

A total of 30 animals were randomly assigned to sham, TxT+H/R_RL or TxT+H/R_EP group (n = 10 per group) for the survival analysis. The sham group was subjected to all surgical procedures without TxT+H/R. For the molecular analyses the following group sizes are given, sham: n = 6, TxT+H/R_RL: n = 8 and TxT+H/R_EP n = 8.

### Blood gas analyses

Five blood gas analyses were performed during the experimentation period. The first samples were taken after cannulation of the femoral artery and before the induction of chest trauma (baseline), the second analysis followed after surgical preparation (post TxT), the next two analyses were carried out at the end of hemorrhagic shock phase (post H) and at the end of reperfusion phase (post R), respectively. Finally, the last blood gas analysis was performed during sacrifice (2 h post R). sO_2_ (%), base excess (mmol/L), and haemoglobin (tHb) were examined using GEM Premier 4000 (Instrumentation Laboratory GmbH, Kirchheim, Germany; BGA Set Optimedical Comfort Sampler Basic Kit).

### Examination of liver injury

Plasma samples were stored at -80°C for later analysis of alanine aminotransferase (ALT) using the the Spotchem EZ SP-4430 device (Arkray, Japan). Determination of the histological liver damage was performed observing for relevant lesions (e.g. congestion, hemorrhage, necrosis, inflammation) among experimental groups in a blinded manner by an independent examiner.

### Ribonucleic acid (RNA) isolation, semi-quantitative reverse transcription-polymerase chain reaction (qRT-PCR)

Total RNA of snap-frozen liver samples was isolated using the RNeasy-system (Qiagen, Hilden, Germany) according to the manufacturer’s instructions. The RNase-Free DNase Set was used to remove the residual amounts of remaining DNA according to the manufacturer`s instructions (Qiagen, Hilden, Germany). The RNA samples were stored at -80°C. For qRT-PCR 100 ng of total liver RNA were reversely transcribed using the Affinity script QPCR-cDNA synthesis kit (Stratagene, La Jolla, CA, USA) following the manufacturer`s instructions. The analysis of the mRNA expression of *IL-6* and *ICAM* was carried out on a Stratagene MX3005p QPCR system (Stratagene) using gene-specific primers for rat *IL-6* (NM_012589, UniGene#: Rn.9873, Cat#: PPR06483B) and rat *ICAM* (NM_012967, UniGene#: Rn.12, Cat#: PPR42235A) purchased from SABiosciences (SuperArray, Frederick, MD, USA). The expression of *gapdh* with rat Gapdh (NM_017008, UniGene#: Rn.91450, Cat#: PPR06557A, SABiosciences, SuperArray, Frederick, MD, USA) was measured as reference. The PCR reaction was set up with 1x RT^2^ SYBR Green/Rox qPCR Master mix (SABiosciences) in a 25 μl volume according to manufacturer`s instructions and as described before [33,34]. Relative expression of each target genes mRNA level was then calculated using the comparative threshold-cycle (CT) method (2^_ΔΔCT^ method). Here, the amount of target mRNA in each sample was normalized to the amount of *gapdh*, to give ΔCT, and then to a calibrator consisting of samples obtained from the sham group. The relative mRNA expression of target genes is presented as % calculated in relation to 100% sham after normalization to *gapdh* [35].

### Detection of polymorphonuclear leukocytes

Analysis of the liver infiltration with PMNL was performed by chloroacetate esterase staining (CAE, 4% pararosanilin, 4% sodium nitrite and naphthol solution) for 30 min at room temperature (RT) as described previously [9]. All sections were counterstained with hematoxylin. The number of infiltrating PMNL was quantified by counting CAE-positive cells in a total of 20 high power (400 x) fields per section per animal in a blinded manner. Data from each tissue section were pooled to determine mean values.

### Staining of ICAM and HMGB1

Cryosectioned liver samples (3 μm) were air-dried for 10 min, fixed in acetone 10 min at RT and air-dried for 60 min at RT. Sections were washed with PBS and water. Then endogenous peroxidase activity was blocked with hydrogen peroxide in accordance with manufacturer's instructions (Peroxidase UltraVision Block, Dako, Hamburg, Germany). The sections were washed and subsequently, mouse anti-rat CD54 monoclonal antibody (BD Pharmingen, Heidelberg, Germany) diluted 1:150 in Antibody Diluent with Background Reducing Components was used as primary antibody (Dako) (room temperature, RT). Anti-mouse horseradish peroxidase-linked secondary antibody (30 min, RT, Simple Stain Rat MAX PO, Nichirei Biosciences Inc., Japan) and 3-amino-9-ethylcarbazol (AEC, DCS Innovative Diagnostik-Systeme, Hamburg, Germany) were used to detect specific binding. Sections were counterstained with hematoxylin.

Paraffin-embedded sections of liver samples (3 μm) were deparaffinized, rehydrated, and stained with polyclonal antibody against HMGB1. After deparaffinization antigen retrieval was performed using Target Retrieval Solution Citrate pH6 under steam atmosphere (Dako) for 20 min (Retriever 2010, Prestige Medical). Then, endogenous peroxidase activity was blocked with hydrogen peroxide in accordance to manufacturer's instructions (Peroxidase UltraVision Block, Dako). Rabbit antibody against HMGB1 (Abcam, 1 μg/ml) in Antibody Diluent with Background Reducing Components was used as primary antibody (Dako). Anti-rabbit horseradish peroxidase-linked secondary antibody (Simple Stain Rat MAX PO (R), Nichirei Biosciences Inc.) and AEC (DCS Innovative Diagnostik-Systeme) were used to detect specific binding. Sections were counterstained with hematoxylin.

### Determination of NF-κB activation

NF-κB p65 ELISA kit (Enzo, Lausen, Switzerland) was applied to measure the active form of NF-κB p65 in liver protein extracts. The working buffer was prepared, and the wild type or competitor duplex were added in the wells as suggested by the provider. Afterwards, liver protein extract (40 μg protein) or TNF-alpha-activated HeLa cell nuclear extract serving as positive control were added. After the short incubation period of one minute, the washing procedure was followed by adding 100 μl of diluted NF-κB p65 antibody (provided in the kit). The samples were incubated for one hour. Then, the washing step was repeated and 100 μl of the horseradish peroxidase-conjugated secondary antibody were added in each well. After one hour, the wells were washed and 100 μl chemiluminescent substrate were added. The extinction was captured after 15 min as suggested by the manufacturer and the percentage of active NF-κB p65 related to the control (sham) is calculated.

### Statistical analysis

One-way analysis of variance (ANOVA) using Kruskal-Wallis with Dunn`s post-hoc test was applied to study the differences between the groups. Changes in target gene expression were analysed by Wilcoxon matched-pair analysis. Data are given as mean ± standard error of the mean (s.e.m.). A p-value below 0.05 was considered statistically significant. All statistical analyses were performed using GraphPad Prism 6 (Graphpad Software, Inc., San Diego, CA).

## Results

All relevant data have been deposited to Figshare: https://figshare.com/articles/Ethyl_pyruvate_ameliorates_hepatic_injury_following_blunt_chest_trauma_and_hemorrhagic_shock_by_reducing_local_inflammation_NF-kappaB_activation_and_HMGB1_release/5616922

### Hemodynamic characteristics and total protein in plasma

**Fig 1:**
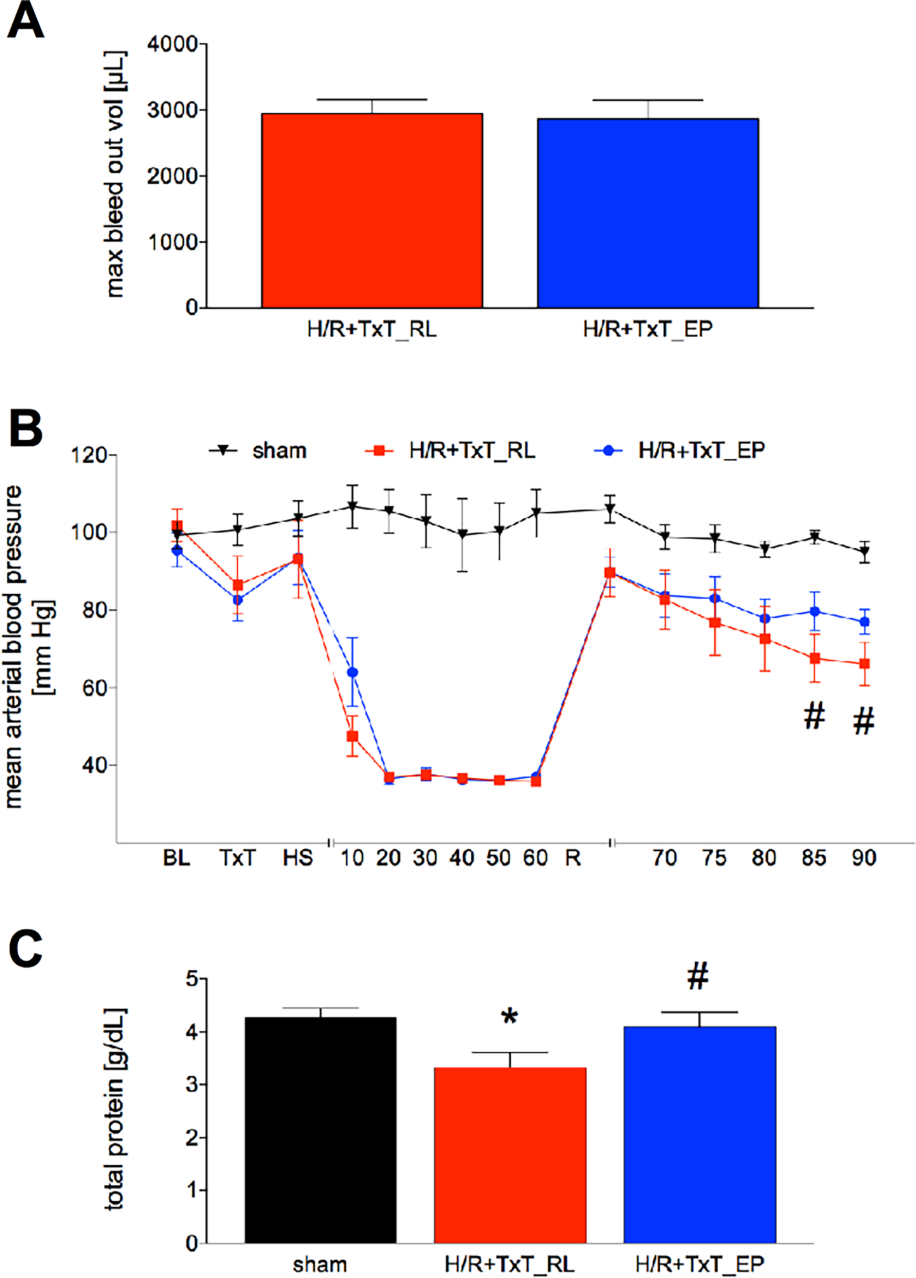
Maximal bleed out volume to induce and maintain hemorrhagic shock (A). Mean arterial blood pressure (MABP) values of all groups during experimentation period, MABP was measured continuous during experimentation. Sham group underwent all surgical procedures without induction of TxT+H/R (B). Total plasma protein values for all groups. Time point: two hours after end of the experiment, sacrifice was performed, and the sample removed (C). Baseline (BL); blunt chest trauma (TxT); ethyl pyruvate (EP); hemorrhagic shock (HS); hemorrhagic shock and resuscitation (H/R); ringer`s lactated solution (RL). *: p <0.05 *vs*. both other groups; #: p <0.05 *vs*. TxT+H/R_RL. Sham: n = 6, TxT+H/R_RL: n = 8 and TxT+H/R_EP: n = 8.

To assess, if there were differences in blood volume to induce hemorrhagic shock that may affect the results, this was evaluated at first. The required maximal bleed out volume to induce and maintain hemorrhagic shock did not differ between both experimental groups with and without ethyl pyruvate (Fig 1A). Also, the initial development of the mean arterial blood pressure (MABP) curve was comparable in both trauma groups (Fig 1B). Both TxT+H/R groups developed a similar blood pressure decrease immediately after TxT, which was followed by a subsequent recovery until the beginning of the hemorrhagic shock (Fig 1B). During shock there were no differences between the TxT+H/R_RL and TxT+H/R_EP group. At the beginning of the resuscitation period, the MABP recovered to comparable levels in both groups. However, in the later resuscitation phase the TxT+H/R_EP group achieved a significantly higher MABP compared to the TxT+H/R_RL group (TxT+H/R_RL: 66.14 ± 5.64 *vs*. TxT+H/R_EP: 78.71 ± 3.19, p<0.05, Fig 1B).

In the next step, total protein content in the plasma was evaluated to illustrate the impact of TxT+H/R on the synthesis performance. The total protein content in the plasma was significantly decreased in the TxT+H/R_RL group compared to sham as well as to TxT+H/R_EP group (TxT+H/R_RL: 3.32 ± 0.28 g/dL *vs*. sham: 4.27 ± 0.17 g/dL and TxT+H/R_EP: 4.10 ± 0.27 g/dL, respectively, p<0.05, Fig 1C).

### Blood gas analyses

**Fig 2:**
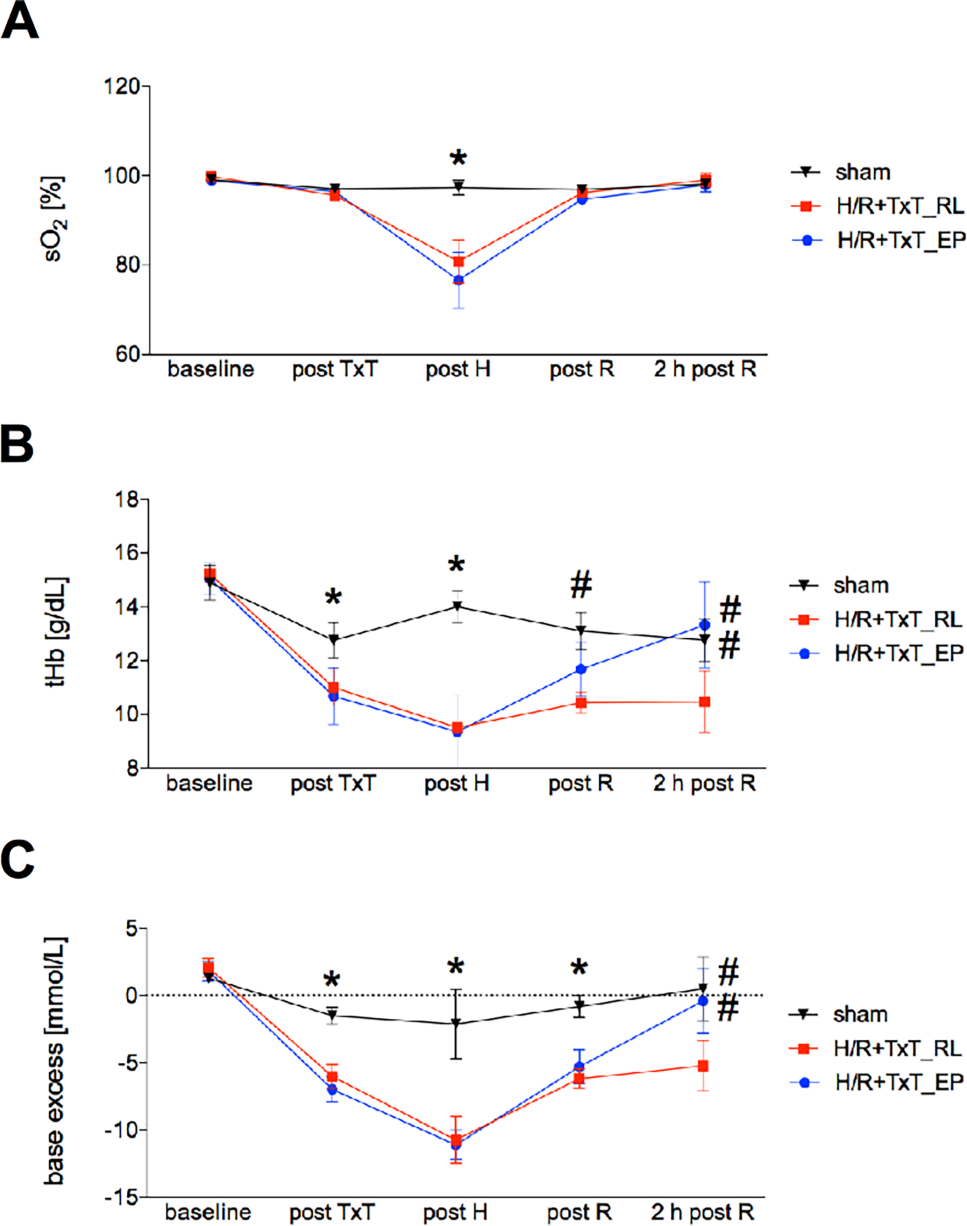
Oxygen saturation (sO_2_, %) values of all groups before the onset of blunt chest trauma (TxT) (baseline), after TxT (post TxT), after hemorrhagic shock (post H), after resuscitation (post R) and at sacrifice (2 h post R). The groups received either ringer`s lactated solution (RL) or ethyl pyruvate (EP) as resuscitation solution after TxT and hemorrhagic shock (TxT+H/R). Sham group underwent all surgical procedures without induction of TxT+H/R. Haemoglobin (tHb) (B) and base excess (C) of all groups during experimentation period are shown. *: p <0.05 *vs*. both other groups; #: p <0.05 *vs*. TxT+H/R_RL. Sham: n = 6, TxT+H/R_RL: n = 8 and TxT+H/R_EP: n = 8.

Beside the above presented hemodynamic monitoring, a continuous control of blood gas analyses for acidosis monitoring was performed. Both TxT+H/R groups showed similar baseline oxygen saturation, a similar decrease after TxT and similar values at the end of hemorrhagic shock, as well as at the end of the resuscitation period or two hours later (Fig 2A).

Both TxT+H/R groups developed a similar total haemoglobin decrease immediately after TxT and hemorrhagic shock (Fig 2B). During this phase, there were no significant differences between the TxT+H/R_RL and TxT+H/R_EP groups. However, there was a significant increase in haemoglobin levels in the TxT+H/R_EP group compared with the TxT+H/R_RL group after resuscitation and at the end of experimentation (TxT+H/R_EP: 13.33 ± 0.81 g/dL *vs*. TxT+H/R_RL: 10.48 ± 1.13 g/dL, p<0.05, Fig 2B).

With regard to base excess, both TxT+H/R groups showed comparable values at baseline time, after TxT, at the end of hemorrhagic shock and at the end of the resuscitation period. However, the TxT+H/R_EP group had significantly higher base excess values at two hours after resuscitation compared with the TxT+H/R_RL group (TxT+H/R_EP: -1.26 ± 1.55 *vs*. TxT+H/R_RL: -5.64 ± 1.26, p<0.05, Fig 2C).

### Liver damage

**Fig 3:**
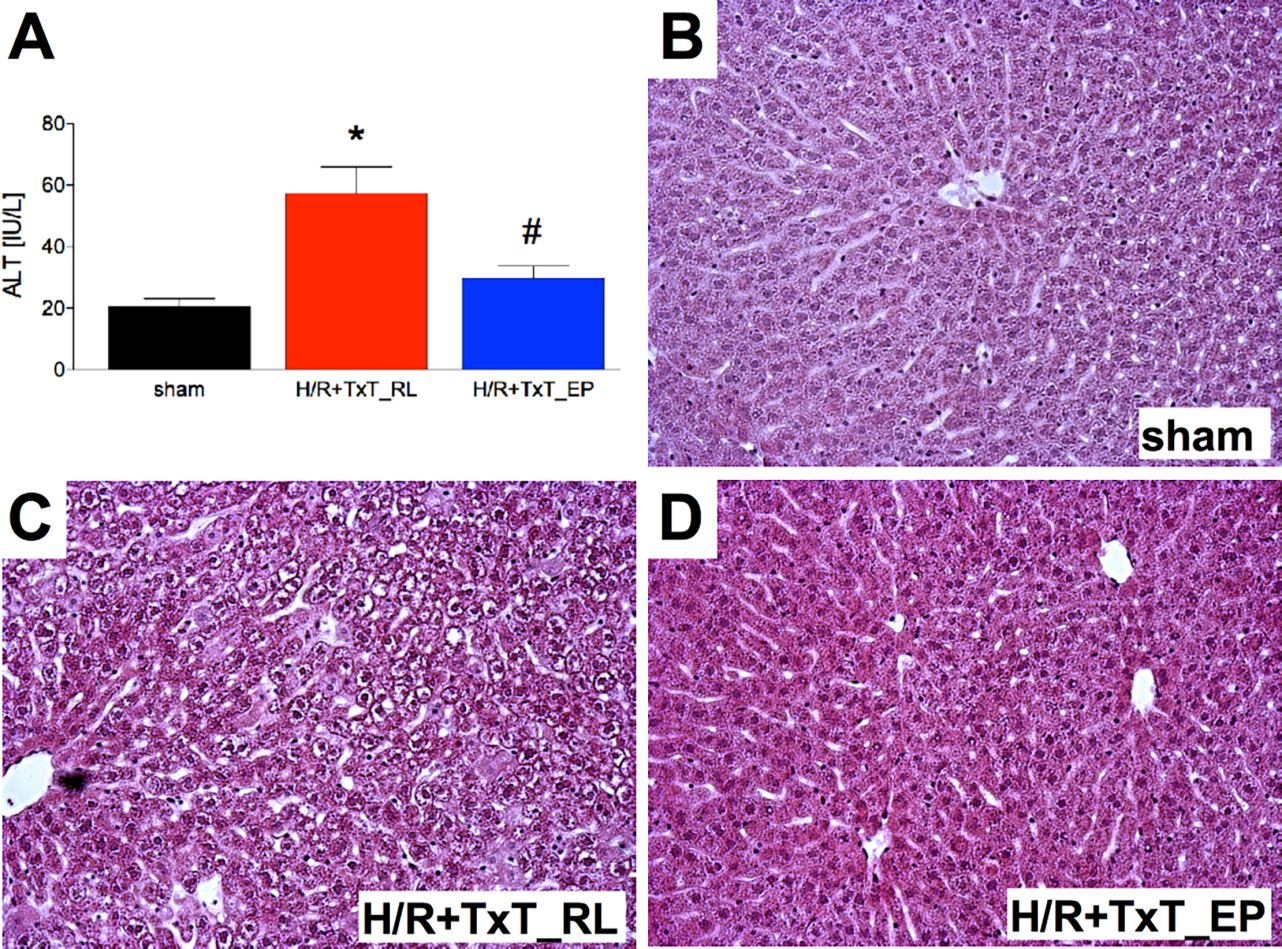
Plasma alanine aminotransferase (ALT) levels of all groups 2 h after resuscitation are shown (A). Sham group underwent all surgical procedures without induction of blunt chest trauma (TxT) and hemorrhagic shock with resuscitation (H/R). The groups received either ringer`s lactated (RL) or ethyl pyruvate (EP) as resuscitation solution after TxT and hemorrhagic shock. *: p <0.05 *vs*. both other groups; #: p <0.05 *vs*. TxT+H/R_RL. Representative hematoxylin and eosin stained liver sections from sham (B), TxT+H/R_RL (C) and TxT+H/R_EP (D) groups are exposed. Sham: n = 6, TxT+H/R_RL: n = 8 and TxT+H/R_EP: n = 8.

After determination of experimental setting and conditions as described above tissue injury and associated inflammatory changes were elaborated. First ALT as a circulating marker of hepatocellular damage was determined. TxT+H/R induced a significant increase of plasma ALT to 57.38 ± 8.59 IU/L at 2 h after resuscitation as compared to 20.46 ± 2.65 IU/L after sham operation (p<0.01, Fig 3A). ALT levels were significantly reduced in the TxT+H/R_EP group (29.63 ± 4.17 IU/L) as compared with the TxT+H/R_RL group (p<0.01, Fig 3A).

In order to verify the changes of liver injury as proposed by ALT levels as described above, histological evaluation of the liver tissue followed. The indicated liver damage by the ALT increase was correspondingly reflected in the histological evaluation. Liver sections from TxT+H/R_RL (Fig 3C) revealed necrosis areas at 2 h after resuscitation compared with sections from either sham (Fig 3B) or TxT+H/R_EP animals (Fig 3D).

### Inflammatory changes in the liver

In the next step the optional key players that are involved in histopathological tissue injury were analysed. To examine the influence of TxT+H/R on the inflammatory system and the postulated anti-inflammatory effects of EP, IL-6 and ICAM gene expression as well as liver infiltration with neutrophils were investigated.

**Fig 4:**
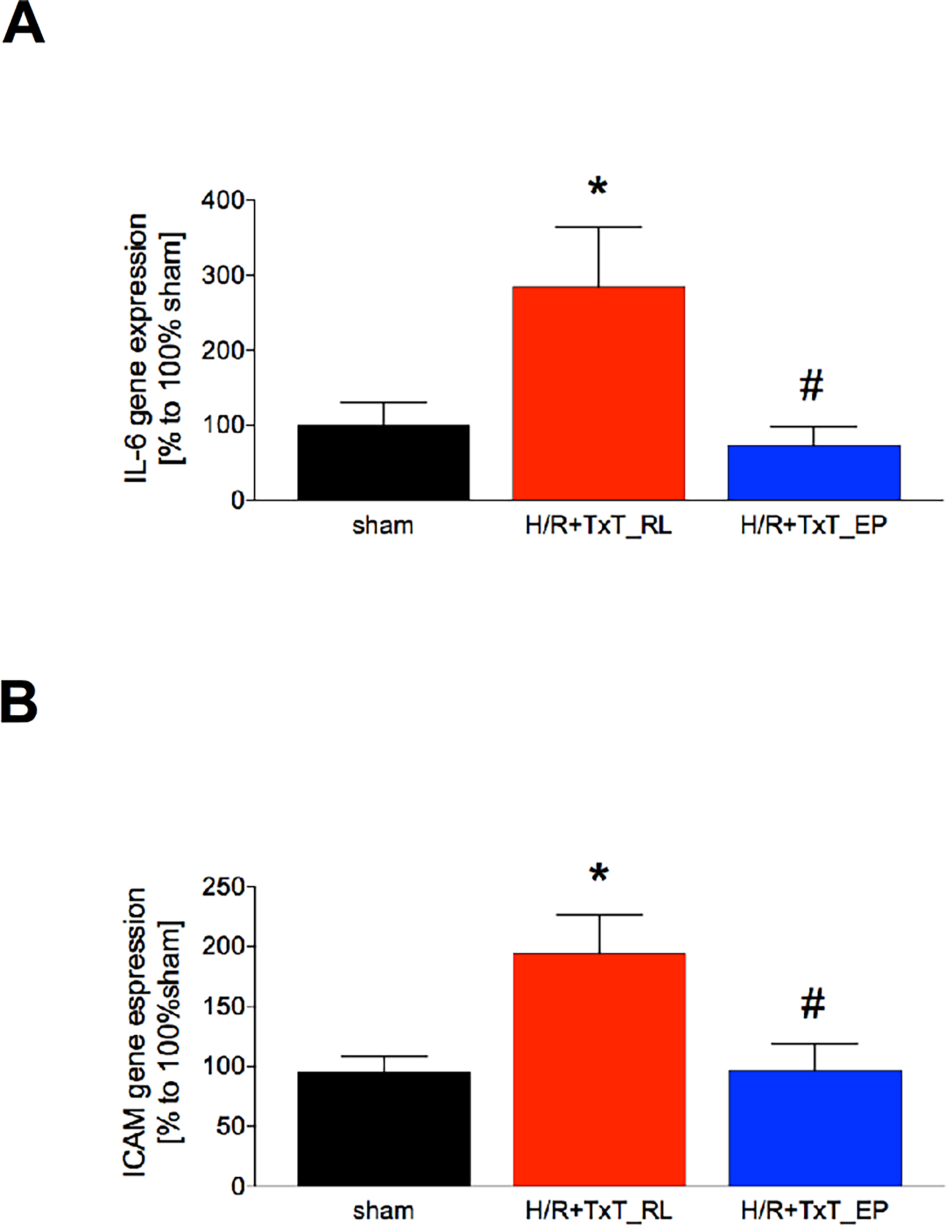
Interleukin (IL)-6 (A) and intercellular adhesion molecule (ICAM)-1 (B) gene expression levels are shown. Sham group underwent all surgical procedures without induction of blunt chest trauma (TxT) and hemorrhagic shock with resuscitation (H/R). The groups received either ringer`s lactated (RL) or ethyl pyruvate (EP) as resuscitation solution after TxT and hemorrhagic shock. *: p <0.05 *vs*. both other groups; #: p <0.05 *vs*. TxT+H/R_RL. Sham: n = 6, TxT+H/R_RL: n = 8 and TxT+H/R_EP: n = 8.

The qRT-PCR showed a significant increase of IL-6 gene expression at 2 h after resuscitation in liver samples obtained from TxT+H/R_RL animals as compared to both other groups (TxT+H/R_RL: 284.2 ± 80.51% *vs*. 100% sham or TxT+H/R_EP: 73.38 ± 25.23%, respectively, p<0.05, Fig 4A). Similar data were found for ICAM gene expression, showing a significant increase at 2 h after resuscitation in TxT+H/R_RL animals as compared to both other groups (TxT+H/R_RL: 193.8 ± 32.43% *vs*. 95.23 ± 13.14% sham or TxT+H/R_EP: 96.78 ± 22.21%, respectively, p<0.05, Fig 4B).

**Fig 5:**
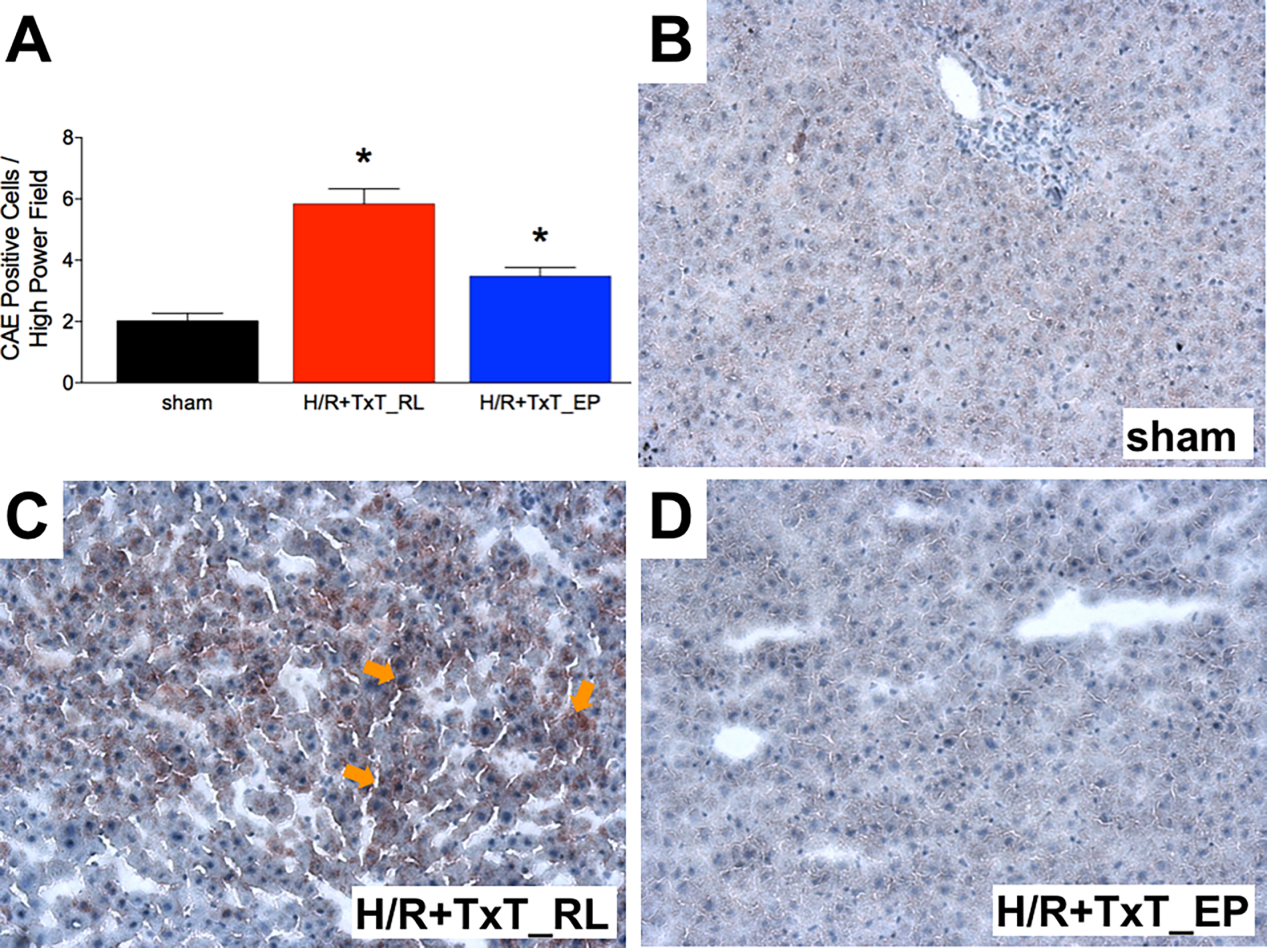
Neutrophils in liver sections were identified by chloroacetate esterase cytochemistry (CAE) and counted in 25 high power fields. Sham group underwent all surgical procedures without induction of blunt chest trauma (TxT) and hemorrhagic shock with resuscitation (H/R). The groups received either ringer`s lactated (RL) or ethyl pyruvate (EP) as resuscitation solution after TxT and hemorrhagic shock. *: p <0.05 *vs*. both other groups. Representative ICAM-1 stained liver sections from sham (B), TxT+H/R_RL (C) and TxT+H/R_EP (D) groups are shown. Sham: n = 6, TxT+H/R_RL: n = 8 and TxT+H/R_EP: n = 8.

Infiltration of the liver with neutrophils significantly increased to 5.84 ± 0.49 cells per high power field at 2 h after resuscitation, as compared to sham animals (2.02 ± 0.25 cells per high power field, p<0.05, Fig 5A). Resuscitation with EP significantly diminished neutrophil infiltration into the liver after TxT+H/R as compared to the TxT+H/R_RL group (3.48 ± 0.28 *vs*. 5.84 ± 0.49 cells per high power field, p<0.05, Fig 5A). Infiltration with neutrophils was markedly increased in the TxT+H/R_EP group compared with sham (p<0.05, Fig 5A).

TxT+H/R markedly increased the ICAM-1 protein expression in the liver in the RL group (Fig 5C) as compared to either sham (Fig 5B) or TxT+H/R_EP group (Fig 5D).

**Fig 6:**
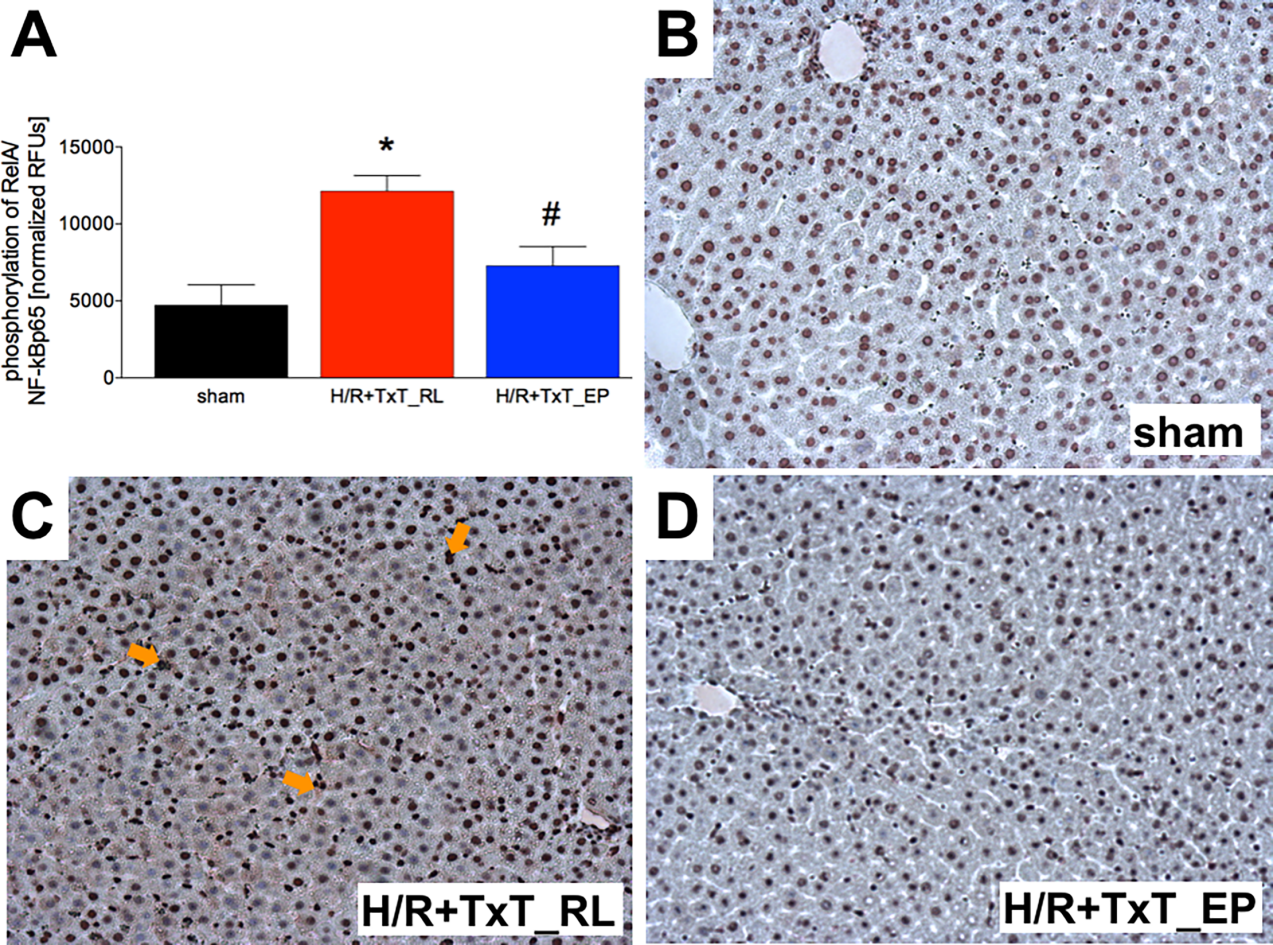
Phosphorylated p65 subunit of NF-κB after normalization as % of the corresponding sham operated group is represented (A). Sham group underwent all surgical procedures without induction of blunt chest trauma (TxT) and hemorrhagic shock with resuscitation (H/R). The groups received either ringer`s lactated (RL) or ethyl pyruvate (EP) as resuscitation solution after TxT and hemorrhagic shock. *: p <0.05 *vs*. both other groups. #: p <0.05 *vs*. TxT+H/R_RL. B-D represents HMGB1 staining in liver sections from sham (B), TxT+H/R_RL (C) and TxT+H/R_EP (D) groups. Sham: n = 6, TxT+H/R_RL: n = 8 and TxT+H/R_EP: n = 8.

After TxT+H/R, HMGB1 staining in liver sections (cytoplasm and cell nuclei) increased compared with either sham-operated or EP-resuscitated animals after TxT+H/R (Fig 6B–6D).

### Analysis of the NF-κB RelA phosphorylation

The proposed mechanism that is involved on the hand in the beneficial impact of EP in inflammatory conditions as shown in other studies intrigues NF-κB. Importantly, NF-κB is generally involved in ICAM-1 and IL-6 expression that was detected in liver samples in the present study. Therefore, to evaluate the mode of action for EP, analysis of phosphorylated RelA/ NF-κB p65 was performed by the NF-κB p65 ELISA kit in liver tissue homogenates, which were collected at 2 h after resuscitation. Analysis of protein content showed significantly increased phosphorylation of RelA/ NF-κB p65 after TxT+H/R in the RL group to 12132 ± 1008 RFU compared to 4725 ± 1215 RFU in sham operated rats (p<0.05, Fig 6A). EP decreased significantly the increase in the amount of phosphorylated RelA/ NF-κB p65 protein as compared to the TxT+H/R_RL group to 7276 ± 1250 RFU (p <0.05, Fig 6A).

### Survival analysis within 24 hours

**Fig 7:**
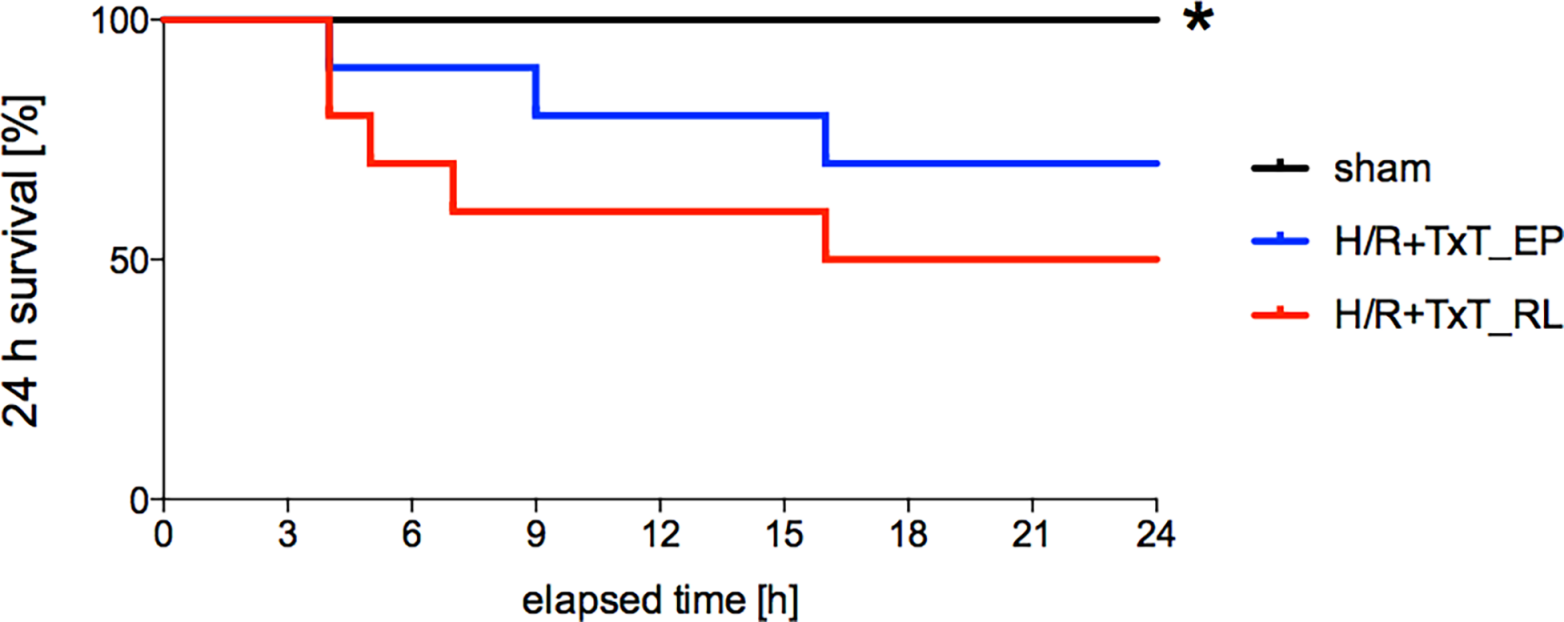
24-hour survival rate of all groups is represented. Sham group underwent all surgical procedures without induction of blunt chest trauma (TxT) and hemorrhagic shock with resuscitation (H/R). The groups received either ringer`s lactated (RL) or ethyl pyruvate (EP) as resuscitation solution after TxT and hemorrhagic shock. A total of 30 animals were randomly assigned to sham, TxT+H/R_RL or TxT+H/R_EP group (n = 10 per group) for the survival analysis.

In the final step, the importance of the tissue-protective findings by EP that were described above on the outcome was elaborated. In order to reveal the effect of using EP as a resuscitation fluid on survival after TxT+H/R, survival at 24 h after trauma was documented. In the sham group, no deaths were observed. TxT+H/R induced a mortality rate of 50% at 24 h in our model. Resuscitating animals with EP largely attenuated the mortality to 30% after TxT+H/R (p = 0.181, Fig 7). These results show that mortality after TxT+H/R was markedly reduced by EP administration in our model.

## Discussion

In the present study, we examined the effect of ethyl pyruvate as resuscitation solution in a double hit model of blunt chest trauma and hemorrhagic shock in rats. Pyruvate is the final product of glycolysis, a scavenger of reactive oxygen species (ROS) and has been tested in numerous *in vitro* and *in vivo* studies [36]. Salahudeen *et al*. showed a beneficial effect of pyruvate regarding renal injury, Mongan *et al*. and Slovin *et al*. found an improved survival after sodium pyruvate infusions in models of hemorrhagic shock, and Sileri *et al*. investigated a protective effect of pyruvate on ischemia/reperfusion injury of the liver [37–40]. The study situation regarding a direct comparison of EP and pyruvate is inconsistent. For example, Zeng *et al*. examined EP and pyruvate in neonatal rat cerebrocortical slices and has found a superiority of EP [41]. On the other hand, Sharma *et al*. detected a advantage of sodium pyruvate compared with EP in a hemorrhagic shock model [42]. Our decision to use EP as a reperfusion solution is mainly based on two important issues. First, pyruvate has a limited stability in solutions, so that in an aldol-like reaction parapyruvate can arise. Second, EP has been used as a safe substance in a Phase II trial in patients with cardiac surgery, although without a significant beneficial effect [43]. The reasons for the results of the Phase II trial remain unclear, but may certainly be caused by the applied dose of EP, selection of the study cohort or no adequate inflammatory response in this setting. However, we believe due to the encouraging *in vitro* and *in vivo* results regarding a potential therapy with EP, that further studies to investigate, understand and use the beneficial effects of EP are required.

Protective effects of ethyl pyruvate in rodents with myocardial ischemia/reperfusion, hemorrhagic or septic shock, or multiple organ injury have already been published before [22,24]. However, the above discussed clinical trial has failed to confirm these effects of ethyl pyruvate in cardiopulmonary bypass [43]. Importantly, models of isolated hemorrhage do not mimic the clinical settings of trauma, and no data are available for the clinically relevant trauma model consisting of blunt chest trauma and hemorrhagic shock. The data from this model show a decrease in haemoglobin, negative base excess values, as well as strong organ injury to the liver, and an increased local inflammatory response after trauma. Ethyl pyruvate as resuscitation solution improved the recovery of haemoglobin and base excess, and reduced the liver damage as shown by reduced ALT values and histopathological damage. Furthermore, ethyl pyruvate reduced the IL-6 and ICAM-1 gene expression which was accompanied with lower infiltration of PMNL into the liver, and furthermore attenuated the release of phosphorylated RelA/ NF-κB p65, as well, indicating at the underlying mechanism.

The effect of trauma and hemorrhagic shock on haemoglobin, oxygen saturation and base excess have been addressed by others before, showing that during the trauma/ hemorrhage phase a drop of haemoglobin and oxygen saturation occurred [44], while the base excess developed negatively, also [45]. Likewise, a recovery in the reperfusion phase is to be expected. Conflictive data have been shown indicating that ethyl pyruvate improved hemodynamics and organ function in porcine endotoxemia [46], as previously reported in rodents as well [47]. In contrast, Dong *et al*. and Mulier *et al*. have shown that ethyl pyruvate failed to improve hemodynamics in splenectomized or nonsplenectomized swine with hemorrhage [48,18]. In our study, the recovery of base excess and haemoglobin in the resuscitation period and two hours later, was more pronounced in the ethyl pyruvate group compared to the control group after trauma/hemorrhage. The higher haemoglobin values in the ethyl pyruvate group compared to the control group also explain the difference with regard to the blood pressure between those two groups at the end of the resuscitation period. Considering that the base deficit is a known indicator for trauma severity, outcome and resuscitation success [45,49–51,12], the better recovery in the ethyl pyruvate group underlines the beneficial potential of ethyl pyruvate as resuscitation fluid. Regarding the total protein content and tHb levels, the TxT+H/R_EP group exhibited higher protein values compared to the TxT+H/R_RL group and slightly lower values compared to the sham group (TxT+H/R_RL: 3.32 ± 0.28 g/dL *vs*. sham: 4.27 ± 0.17 g/dL and TxT+H/R_EP: 4.10 ± 0.27 g/dL, respectively, p<0.05, Fig 1C). However, the total protein content in the plasma was determined at two hours after resuscitation illustrating either the liver recovery or protection by EP during the reperfusion phase. This effect is shown, on the one hand, by the lower liver damage in the TxT+H/R_EP group that has been confirmed and represented in histological evaluation as well. In the TxT+H/R_RL group, we have seen significantly higher liver damage in the histological sections as well as increased ALT values, data suggesting an improved synthesis efficiency of the liver in the EP group as compared to the control after H/R. On the other hand, the small difference between the sham group and the TxT+H/R_EP group in terms of hemodilution is surprising as well as restoration of tHb at 2 hr post resuscitation. However, Yang *et al*. examined in his study the influence of EP on the liver in a setting of severe acute pancreatitis [52]. His group found a significantly higher blood volume in the EP group, while the exact mechanism remained unclear [52]. We did not measure the blood volume between the different groups. Certainly, the blood volume and the underlying mechanism remain to be evaluated in further studies as they may explain the differences in total protein content and restoration of tHb. Trauma severity was comparable in both groups as nicely shown by the comparable base excess values at the end of the hemorrhagic shock period (post H). In line with the improved compensatory mechanisms by applying resuscitation fluid supplemented with ethyl pyruvate after trauma/hemorrhage, mortality has shown a clear trend to a better survival by using this protocol, although the difference was not statistically significant. An increase in the number of animals per group was considered to elaborate a significant difference in terms of survival. However, performing only the survival study without significant molecular analyses was omitted due to ethical reasons and limited resources for only this setting. So, the question of the survival significance should be answered in further larger studies implying additional parameters. In general, then, based on current findings, the endpoints should be chosen more carefully. It may be wise to choose organ or inflammation parameters as primary endpoint, and additionally, if evaluating mortality, the necessary statistical power that may comprise much higher numbers of animals remains to be considered always regarding the ethical aspect as well.

To the best of our knowledge, data regarding the immune modulating effect of pyruvate in a double hit model of blunt chest trauma and hemorrhagic shock are unavailable. However, in other *in vivo* settings, a positive effect of ethyl pyruvate has been shown with regard to the liver and the immune system. Tsung *et al*. exposed rats to 60 minutes of partial warm hepatic ischemia followed by reperfusion with ethyl pyruvate or ringer`s lactated solution [53]. The authors found a decrease of serum transaminase, of degree of hepatic necrosis and of neutrophil infiltration in the liver after treatment with ethyl pyruvate [53]. Moreover, the expressions of hepatic ICAM-1 mRNA and inflammatory cytokines, TNF-α, IL-1β and IL-6, were decreased in ethyl pyruvate treated animals compared with the control group [53]. Yang *et al*. showed in alcohol-intoxicated mice that ethyl pyruvate alleviated the liver damage after abuse [54]. *Inter alia* alanine aminotransferase, histopathological fatty change and necrosis were reduced by ethyl pyruvate [54]. Furthermore, the successful use of ethyl pyruvate for the treatment of liver injury after severe acute pancreatitis has also been confirmed *in vivo* [55]. Also, this experimental design showed a reduction of alanine aminotransferase and histopathological damage [55]. In summary, our data clearly support these findings. With regard to liver damage, in our double hit model of blunt chest trauma combined with hemorrhagic shock, ethyl pyruvate attenuated the increase of alanine aminotransferase and the histopathological liver tissue damage. Moreover, we found in ethyl pyruvate treated animals less gene expression of pro-inflammatory IL-6 and ICAM, reduced levels in ICAM-1 protein expression, lower rates of infiltrating neutrophils into the liver, and less necrosis in the liver.

While the underlying pathomechanism has not been definitively clarified yet, a central role for NF-κB in the regulation of the immune response and apoptosis seems likely [56]. Dong *et al*. have shown that ethyl pyruvate provided therapeutic anti-inflammatory benefits to modulate splenic NF-κB, restraining inflammatory responses and organ injury during resuscitation in lethal porcine hemorrhage [18]. It has been reported further that ethyl pyruvate inhibited LPS-induced activation of p65 NF-κB in RAW macrophages cells, as well as DNA-binding of wild-type p65 NF-κB in HEK293 cells [57,58]. According to these data, our study with blunt chest trauma and hemorrhagic shock induced a substantial increase in activation of RelA/ NF-κB p65. Resuscitation with ethyl pyruvate markedly inhibited RelA/ NF-κB p65 activation in the liver compared with the control group after trauma. This is in line with results of Tsung *et al*., who found an inhibition of NF-κB after warm hepatic ischemia and ethyl pyruvate treatment *in vivo* [53]. Recent studies suggest that ethyl pyruvate protects against coxsackie virus B3-induced acute viral myocarditis by suppression of HMGB1/RAGE/NF-κB pathway [59]. Also with regard to hepatic ischemia/reperfusion injury, it has been demonstrated that inhibition of the intrinsic pathway of apoptosis and autophagy was mediated partly through downregulation of HMGB1/TLR4/NF-κB axis [26]. In our double hit model, blunt chest trauma and hemorrhagic shock induced HMGB1 up-regulation was markedly reduced by ethyl pyruvate. This is in line with the results of Luan *et al*., in his study EP was examined in view of the influence on liver damage in rats with severe acute pancreatitis (SAP) [55]. Similar to our results the increase of inflammatory cytokines, here TNF-α and IL-1β, and also HMGB1 after SAP were attenuated by EP. Regarding the underlying mechanism, Luan *et al*. found decreased NF-κB DNA-binding activity in EP treated animals compared to the control group [55]. Also, *in vivo* in a setting of hepatic fibrosis in rats reduced ALT, AST, IL-6 and HMGB1 values were found after EP treatment compared to the control group accompanied by decreased mRNA levels of TLR4 and NF-κB [60]. Furthermore, a protective effect of EP at alcohol-induced liver injury, hepatic ischemia-reperfusion injury and autoimmune hepatitis has been shown, which has been associated with diminished activation of NF-κB [54,26,61]. Consistent with those findings involving NF-κB, in the study of Shen *et al*. reduced expression of NF-κB in mRNA and protein level was observed [61]. Han *et al*. explored *in vitro* the mechanism of EP and ruled out that the effect was caused by inhibition of IκB degradation or by DNA binding of the p50 subunit of NF-κB [58]. Though, they showed that EP alters Cys^65^ in p65 and thereby blocks the DNA binding [58]. Fink specified the mode of action and reasoned that efficacy of EP is caused by the “NF-kB-dependent signalling by interfering with binding of the transcription factor to cis-acting response elements in the promoter regions of target genes” [22]. In summary, EP shows a multidisciplinary protective effect on the liver, which may be linked to the inhibition of NF-kB DNA binding, and to the best of our knowledge, there are no further studies in a combination model of blunt chest trauma and hemorrhagic shock regarding EP.

In conclusion, our current data suggest that the pronounced local pro-inflammatory response in the liver after blunt chest trauma and hemorrhagic shock is mediated *via* NF-κB. Furthermore, ethyl pyruvate, as a non-toxic compound, can provide therapeutic anti-inflammatory benefits by regulating the liver HMGB1/NF-κB axis, thereby, restraining inflammatory responses, and organ injury after double hit trauma in rats.
